# Early IFN-β administration protects cigarette smoke exposed mice against lethal influenza virus infection without increasing lung inflammation

**DOI:** 10.1038/s41598-022-08066-7

**Published:** 2022-03-08

**Authors:** Wenxin Wu, Lili Tian, Wei Zhang, J. Leland Booth, Jerry William Ritchey, Shuhua Wu, Chao Xu, Brent R. Brown, Jordan P. Metcalf

**Affiliations:** 1 Pulmonary, Critical Care and Sleep Medicine, Department of Medicine, University of Oklahoma Health Sciences Center, Room 425, RP1, 800 N. Research Pkwy., Oklahoma City, OK 73104 USA; 2 Department of Veterinary Pathobiology, College of Veterinary Medicine, Oklahoma State University, Stillwater, OK USA; 3 Division of Geriatrics, The Second Affiliated Hospital of Soochow University, Suzhou, Jiangsu People’s Republic of China; 4 Department of Biostatistics and Epidemiology, University of Oklahoma Health Sciences Center, Oklahoma City, OK USA; 5 Veterans Affairs Medical Center, Oklahoma City, OK USA; 6 Department of Microbiology and Immunology, University of Oklahoma Health Sciences Center, Oklahoma City, OK USA

**Keywords:** Cytokines, Infection, Infectious diseases, Inflammation, Innate immune cells, Innate immunity

## Abstract

During influenza A virus (IAV) infection, it is unclear whether type I interferons (IFNs) have defensive antiviral effects or contribute to immunopathology in smokers. We treated nonsmoking (NS) and cigarette smoke (CS)-exposed mice intranasally with early (prophylactic) or late (therapeutic) IFN-β. We compared the mortality and innate immune responses of the treated mice following challenge with IAV. In NS mice, both early and late IFN-β administration decreased the survival rate in mice infected with IAV, with late IFN-β administration having the greatest effect on survival. In contrast, in CS-exposed mice, early IFN-β administration significantly increased survival during IAV infection while late IFN-β administration did not alter mortality. With regards to inflammation, in NS mice, IFN-β administration, especially late administration, significantly increased IAV-induced inflammation and lung injury. Early IFN-β administration to CS-exposed mice did not increase IAV-induced inflammation and lung injury as occurred in NS mice. Our results demonstrate, although IFN-β administration worsens the susceptibility of NS mice to influenza infection with increased immunopathology, early IFN-β administration to CS-exposed mice, which have suppression of the intrinsic IFN response, improved outcomes during influenza infection.

## Introduction

Cigarette smoking is the leading cause of preventable death in people. It is the primary cause of chronic obstructive pulmonary disease (COPD), and predisposes these subjects to severe respiratory tract infections^1^. Influenza A virus (IAV) is one of the most frequent triggers of COPD exacerbations which are a major cause of hospital admissions^2^. IAV infection in general is a major cause of infectious morbidity and mortality, and CS exposure alone is associated with more frequent and severe infections with IAV.

The innate immune system is the first line of defense against infection. To initiate productive infection and to cause disease, IAV must overcome host defenses. The innate responses to IAV are triggered by recognition of pathogen associated molecular patterns by three families of pattern recognition receptors (PRRs): Toll-like receptors (TLRs), RIG-I like helicases (RLRs) and nucleotide-binding domain and leucine-rich-repeat-containing proteins (NLRs). PRRs are capable of recognizing IAV and initiating the antiviral innate immune response. They do so through antiviral interferon (IFN) and proinflammatory cytokine responses in order to contain the infection^3^. The IFN responses are central to antiviral immunity against viral infection. IFNs are further divided into type I (mainly IFN-α and β), II (IFN-γ) and III (IFN-λ) subtypes. When an IFN interacts with its unique cognate receptor, a signal is rapidly transmitted within the cell, leading to increased expression of interferon-stimulated genes (ISGs) and augmented antiviral activity.

We have shown that antiviral responses are suppressed by cigarette smoke (CS) exposure^4,5^. Specifically, we demonstrated that RIG-I and IFN-β mRNA induction by IAV were inhibited by CS in human cells and animals. CS exposure increased mortality in wild type (WT) mice infected with IAV. RIG-I overexpression in mice protects against CS-enhanced susceptibility to influenza infection^6^. Our recent data showed that deletion of RIG-I did not change survival rates or innate immune responses in vivo during IAV infection in mice^7^. The results suggest that suppression of multiple PRRs by CS in addition to RIG-I is required for functional immunosuppression.

During influenza infection, type I IFNs can have beneficial antiviral effects or can worsen immunopathology, depending on the strain of IAV and the individual host^8^. Results from two research groups found administration of IFN-α protected animals from lethal challenge with IAV^9,10^. However, excessive type I IFN signaling or prolonged IFN administration during acute influenza infection may increase mortality^11^. In vitro*,* prophylactic exogenous IFN-β is effective to improve outcomes of IAV and highlights the potential for repeated doses to patients at risk during viral seasons of infection^12^.

Influenza infection is not the only viral illness for which timing of the IFN response appears to play an important role in the consequences of disease. In Middle East respiratory syndrome–coronavirus (MERS-CoV), the relative timing of the type I IFN response is a key in determining outcomes in an animal model^13^.

In this study, we focus on influenza infection. We wished to specifically address whether timing of IFN administration during influenza infection affected outcomes, and whether these effects were altered in the presence of suppression of the innate antiviral response by CS. We report that early and late IFN administration to normal nonsmoking (NS) mice worsened inflammation, acute lung injury (ALI), and mortality. In contrast, early IFN administration to CS-exposed mice enhanced the host PRR response to IAV infection and decreased mortality.

## Results

### IFN-β administration is detrimental for survival in lethal IAV infection in nonsmoking (NS) mice

**Figure 1:**
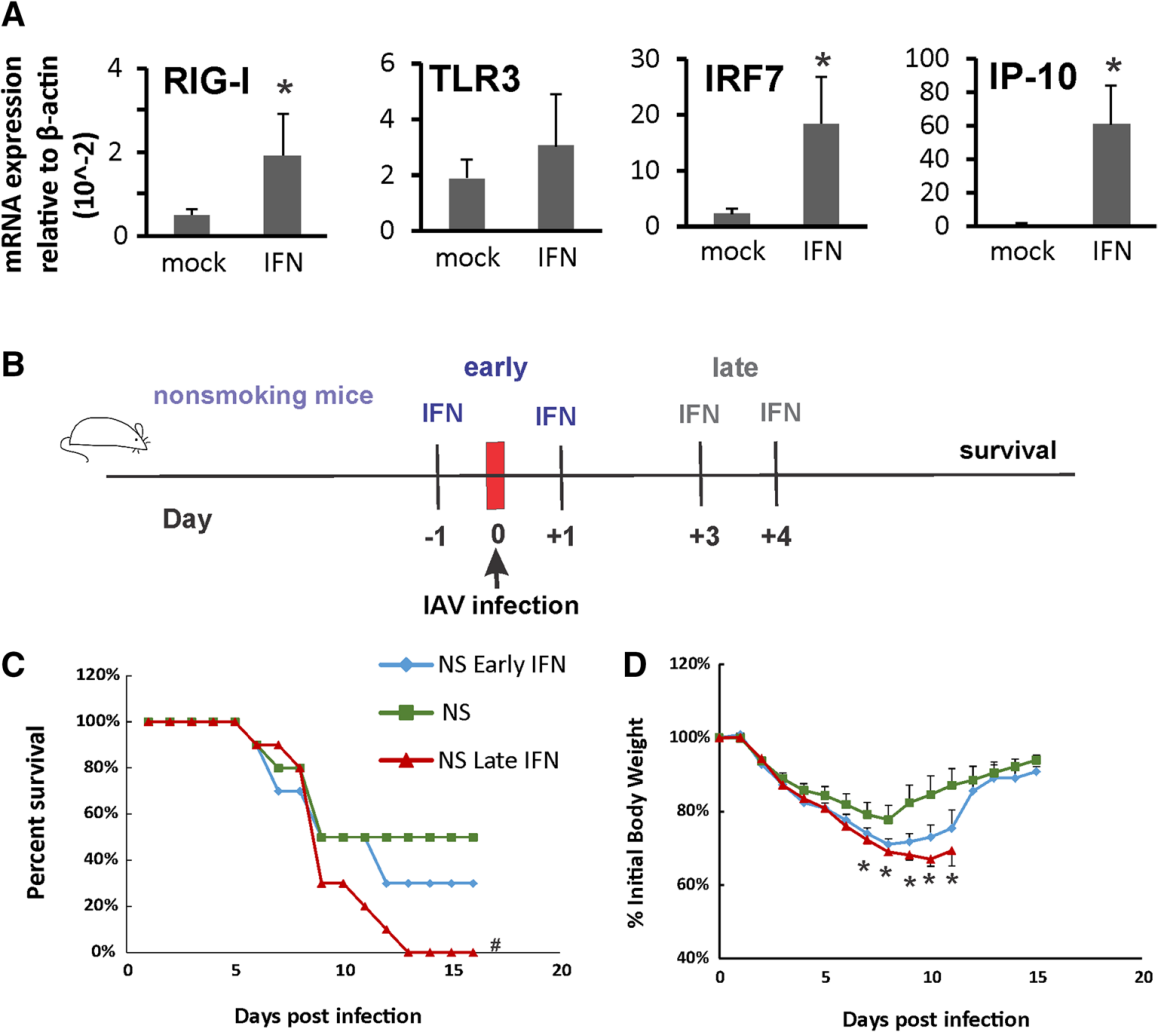
IFN-β administration is detrimental for survival in lethal IAV infection in nonsmoking (NS) mice. (**A**) IFN-β administration induced robust innate immune responses in mouse lung. C57BL/6 mice were administrated with IFN-β (2000 U) intranasally in a total volume of 50 μl in 0.1%BSA/PBS. After 6 h, mouse lungs were collected. mRNA levels were assessed by qRT-PCR and normalized to β-actin. Bar graph represents mean ± standard deviation (n = 3). (**B**) Schematic of the experimental plan on early or late IFN-β administration and IAV infection to NS mice. Mortality (**C**) and body weights (**D**) during lethal IAV infection in IFN-β administrated NS mice. The mice treated with IFN-β (2000 U) intranasally either early (at 1 day before and 1 day after IAV infection) or late (at day 3 and 4 after IAV infection) in a total volume of 50 μl in 0.1%BSA/PBS. The mice were intranasally inoculated with IAV at 1000 PFU/mouse. Mortality and body weights were monitored daily. Body weight data were normalized to each mouse’s starting body weight. Data are expressed as mean ± standard deviation (n = 10 for all groups). # denotes significant survival rate difference between the NS and NS-Late IFN groups, *p* < 0.05. *Denotes significant weight loss difference between the NS and NS-Late IFN groups, *p* < 0.05.

First, we confirmed that IFN-β administration induced innate immune responses in non-infected animals. Mice were lightly anesthetized with isoflurane and treated with IFN-β (2,000 U) intranasally in a total volume of 50 μl in 0.1%BSA/PBS. Mock groups were sham treated with a single dose of intranasal sterile 0.1%BSA/PBS solution (diluent). We found that IFN-β induced robust innate immune responses in the mouse lung, including increased mRNA of RIG-I and the downstream transcription factor IRF7. Significantly, mRNA levels of interferon gamma-induced protein 10 (IP-10, a cytokine downstream of RIG-I and IFN-β) were induced 150-fold over mock (Fig. 1A).

In order to determine whether IFN administration improves mortality in NS mice, we treated the mice intranasally with early IFN-β (NS-E) or late IFN-β (NS-L). In the early IFN group, mice were given IFN-β at 1 day before and 1 day after IAV infection (Fig. 1B). We did not observe weight loss in IFN-β treated animals prior to influenza infection. In the late IFN group, mice were given IFN-β at day 3 and 4 after IAV infection. In the NS group, mice were given sterile diluent at day 1 and 3 after IAV infection. We inoculated IFN-β treated or diluent treated animals with a lethal dose of virus (1000 PFU). This dose was selected to cause approximately 50% mortality (Lethal Dose, 50%; LD_50_) in NS mice. Death was recorded when mice were found dead in the cage or at 70% of original body weight. The mock group, which was sham inoculated with an equal volume of PBS, had no mortality and weight loss (not shown). Late IFN administration to NS mice significantly increased overall mortality during lethal dose viral infection by Fisher’s exact test (Fig. 1C). Early IFN administration to NS mice also appeared to increase mortality although the survival curve was not significant different. Specifically, early and late IFN administration had survival rates (NS Early IFN 30%; NS Late IFN 0%) compared to diluent treated animals (NS, 50%, n = 10 all groups). While mortality in the diluent treated group plateaued at 9 days post infection (p.i.), additional mortality occurred after this time p.i. in the IFN treatment groups, especially in NS Late IFN group (Fig. 1C). Weight loss occurred in all infected groups (Fig. 1D). The body weight data correlated with the survival data in that the lower survival groups lost more weight than groups with higher survival. Accordingly, the maximum body weight loss of IFN administration groups infected with IAV (29% and 31% loss for NS Early IFN and NS Late IFN respectively) was significantly greater than in the IAV infected diluent treated group (NS, 22% loss; Fig. 1D).

### Early IFN-β administration improves survival in CS-exposed mice during lethal IAV infection

**Figure 2:**
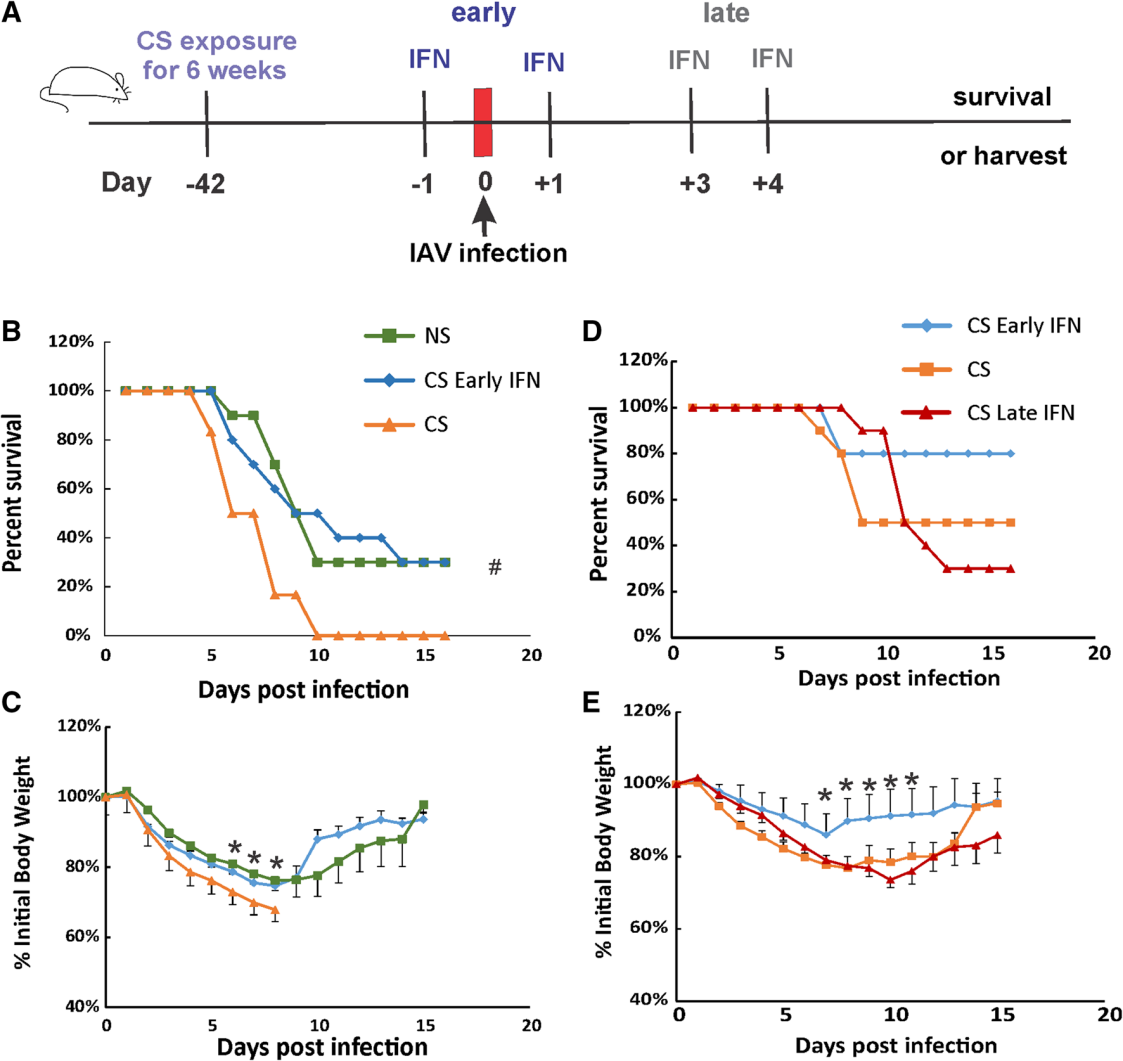
Early IFN-β administration increased survival rates of CS-exposed IAV-infected mice. (**A**) Schematic of the experimental plan on CS exposure, early or late IFN-β administration and IAV infection. Mortality (**B**) and body weights (**C**) during lethal IAV infection (1000 PFU/mouse) in IFN-β administrated mice. Mortality (**D**) and body weights (**E**) during lethal IAV infection (500 PFU/mouse) in IFN-β administrated CS mice. The mice were exposed to CS for 6 weeks. Then, the mice were treated with IFN-β (2000 U) intranasally either early (at 1 day before and 1 day after IAV infection) or late (at day 3 and 4 after IAV infection) in a total volume of 50 μl in 0.1%BSA/PBS. Mortality and body weights were monitored daily. Body weight data were normalized to each mouse’s starting body weight. Data are expressed as mean ± standard deviation (n = 11 for CS group in B and C, n = 10 for other groups). # denotes significant survival rate difference between the CS and CS-Early IFN groups, *p* < 0.05. * denotes significant weight loss difference between the CS and CS-Early IFN groups, *p* < 0.05.

We have shown that CS exposure increased morbidity and mortality of IAV infection in mice^14^. We next evaluated the effect of IFN-β administration on mortality and weight loss during IAV infection in CS-exposed mice. Whole-body CS exposure was performed as described^5^. Briefly, mice were exposed to CS for 4 h per day for 6 weeks. Exposure was accomplished using a smoking chamber (Teague Enterprises, Davis, CA). After 6-week CS exposure, some of the mice were treated intranasally with IFN-β administration. All mice were inoculated with a lethal dose of PR8 virus (Fig. 2A). In Fig. 2B, C, after IAV infection (1000 PFU/mouse), CS-exposed mice had a lower survival rate than NS mice (0% for CS vs. 30% for NS mice). However, by Fisher’s exact test, early IFN-β administration to CS-exposed mice (CS Early IFN) significantly improved survival compared to diluent treated mice during IAV infection (30% survival for CS Early IFN group vs. 0% for CS group, *p* < 0.05). Morbidity was also decreased by early IFN-β administration, as early IFN-β-treated mice had less weight loss compared diluent treated mice (Fig. 2B). Thus, early IFN-β administration significantly reduced weight loss and improved survival in CS-exposed mice during IAV infection. We did not include the CS-Late IFN mice in the 1000 PFU/mouse dosage as we had already determined that late IFN administrated significantly worsened survival in NS animals. Thus, we would be unlikely to see an improvement in survival in these animals, particularly with the high mortality rate of untreated 1000 PFU exposed CS animals. This was confirmed in subsequent experiments using lower viral doses (see below).

Next, we experimentally determined an LD_50_ for CS-exposed mice and discovered this dose was 500 PFU/animal. We used this dose in the next survival test on CS-exposed mice with or without IFN-β administration (Fig. 2D, E). As in CS mice infected with 1000 PFU/animal, early IFN-β administration improved survival compared to diluent treated mice at the LD_50_ for CS-exposed mice (80% for CS Early IFN vs. 50% for CS mice). Late IFN-β administration, on the contrary, decreased survival compared to diluent treated CS mice (30% for CS Late IFN vs. 50% for CS mice). The body weight data correlated with the survival data in that the highest survival group (CS Early IFN) lost significantly less weight than groups with lower survival. The data suggested that early IFN-β administration improved, while late IFN-β administration worsened, morbidity and mortality in CS-exposed mice during IAV infection.

### IFN-β administration increased inflammatory response in NS mice but not in CS-exposed mice during IAV infection

To investigate the role of IFN-β administration in the inflammatory response to IAV, mice were inoculated intranasally with IAV at 500 PFU/mouse. The mock group was sham inoculated with an equal volume of PBS as a negative control. Animals were sacrificed by overdose isofluorane at 5 days p.i.

**Figure 3:**
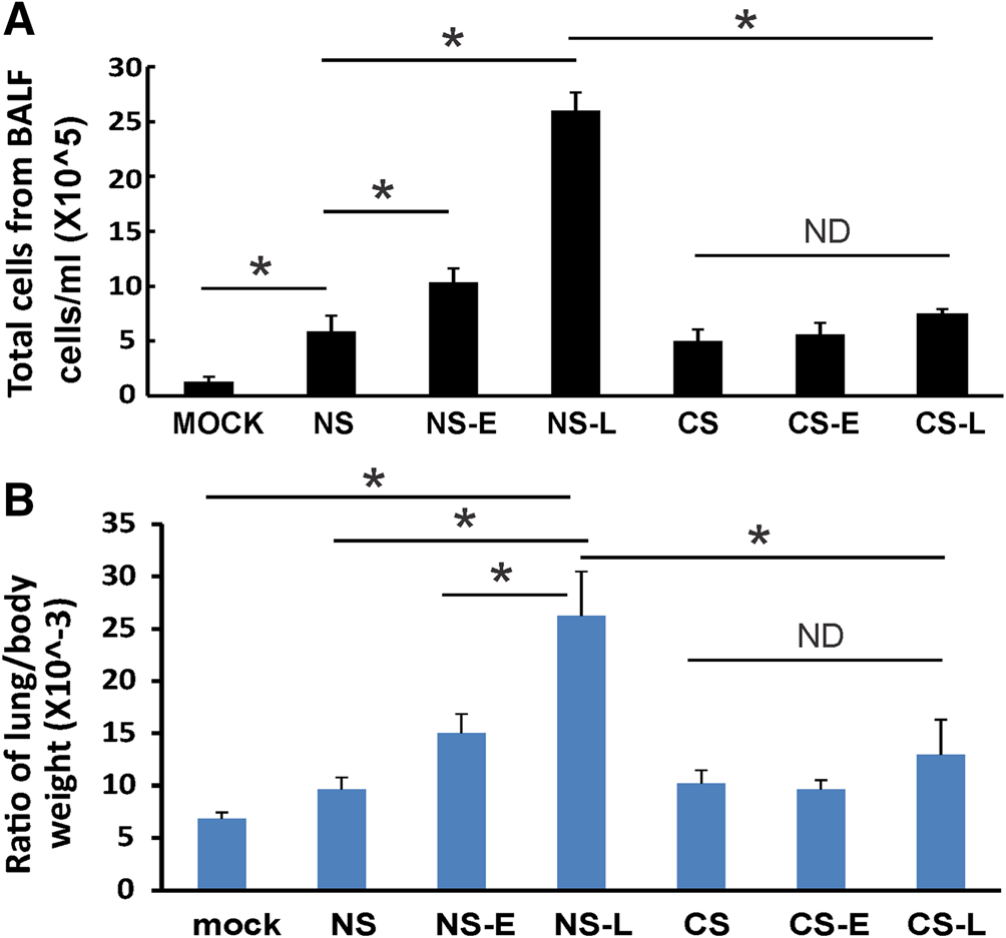
Lung injury and cellularity in the lungs. CS exposure, early or late IFN-β administration and IAV infection are the same as Fig. 2A. Each mouse was infected with 500 PFU of IAV. Mock treated mice were inoculated with PBS. Bronchoalveolar lavage fluid (BALF) or lung tissue was harvested at day 5 after infection. Total immune cells (**A**) in BALF and ratio of lung/body weight (**B**) were determined. Data are expressed as means ± SEM (n ≥ 4/group). ^*^denotes significant difference between the two groups, *p* < 0.05. ND = no significant difference between the two groups.

We first determined the total inflammatory cell numbers in bronchoalveolar lavage fluids (BALF). IAV inoculation increased the total viable leukocytes in BALF in mice. Both early (NS-E) and late (NS-L) IFN-β administration to NS mice significantly increased the total BAL cell numbers with late IFN-β administration showing the highest total BAL cell counts during the infection (Fig. 3A). CS exposure decreased the total BALF cell number during IAV infection (CS vs. NS) as we have previously shown^6^. Both early and late IFN-β administration to CS mice (CS-E and CS-L, respectively) did not increase total BALF cell numbers as was seen in NS-E and NS-L groups. These results showed that IFN-β administration increased immune cell influx into the lung in NS mice but not in CS-exposed mice during IAV infection.

IAV induced lung injury was characterized by examining the lung-to-body weight ratio. The lung-to-body weight ratio is an indicator for ALI and is also an indirect measure of the lung inflammatory response^15,16^. IAV infection of mice significantly increased lung-to-body weight ratio (NS vs. Mock, *p* < 0.05, Fig. 3B). Early or late IFN-β administration to NS mice also increased the ratio with late IFN-β administration (NS-L) causing a significant increase in the ratio over induced by infection alone (NS vs. NS-L, *p* < 0.05, Fig. 3B). In contrast, IFN-β administration to CS-exposed mice did not increase the ratio as seen in NS mice after infection (Fig. 3B). Thus, IFN-β administration increased lung injury and cellular influx in NS mice but not in CS-exposed mice during IAV infection.

### Effect of IFN administration on lung histopathology during IAV infection

**Figure 4:**
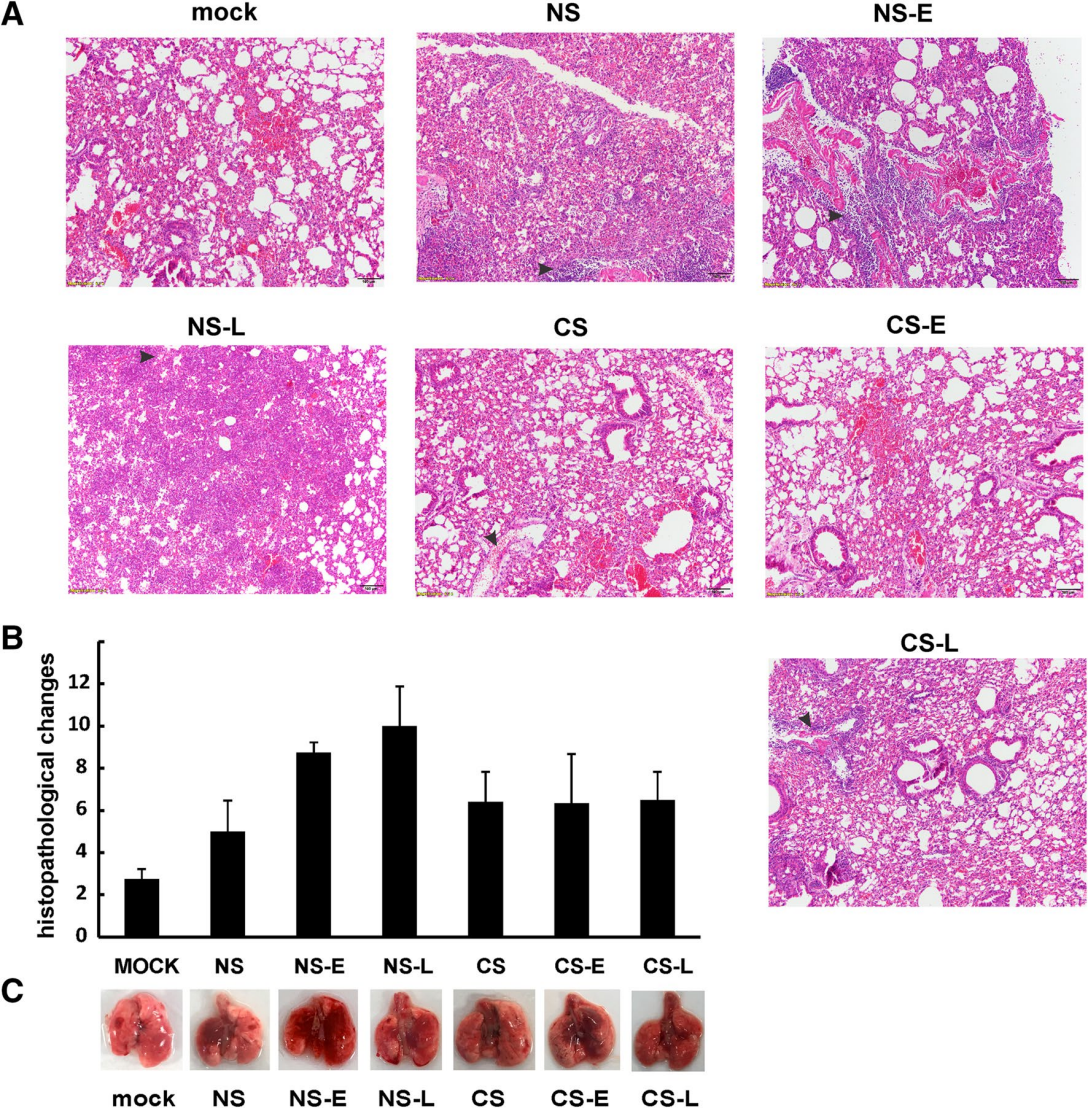
Mouse lung tissue pathology after IAV infection. CS exposure, early or late IFN-β administration and IAV infection are the same as Fig. 2A. Each mouse was infected with 500 PFU of IAV PR8. Animals were sacrificed at 5 days after infection and lung tissue was harvested. Lung tissue sections prepared from the infected mice were fixed, processed and stained with hematoxylin–eosin (**A**). Histopathologic evaluation and scoring of IAV infection were determined by a blinded pathologist (**B**). The lungs of 5 mice from each treatment group were processed for histology, and results shown were typical for the group. The arrows indicate infiltrated neutrophils, edema or hemorrhage. (**C**) Gross pathologic images of IAV-infected lungs. The image shown is representative of 5 mice lungs from each treatment group.

The IAV-infected groups displayed a typical histopathological pattern for viral pneumonia, including severe bronchi and bronchioles inflammation characterized by epithelial cell necrosis and sloughing with partial to complete airway obstruction by intact and degenerate neutrophils and cellular debris, and varying degrees of acute intra-alveolar edema and/or hemorrhage (Supplemental Fig. 1). IAV infection also resulted in the expected neutrophilic alveolar infiltrate with some lymphocytes (Supplemental Fig. 1C and D). Histopathologic scoring of the cardinal features of IAV infection (Fig. 4A) was evaluated as in a previous publication by a pathologist (JWR) blinded to the treatment groups^17^. Consistent with the total inflammatory cells and lung-to-body ratio data, early or late IFN-β administration to NS mice appeared to result in higher overall pathologic scores than other groups with NS-L having the highest pathologic score among all groups (Fig. 4B). Scores in the CS group were higher than in the NS group. In contrasts to the effects seen in the NS groups, IFN-β administration to CS mice caused no difference in the severity of inflammation in these mice. From gross observation, we found that lungs from NS-E, NS-L and CS-L mice had the most prominent lung hemorrhage among the groups (Fig. 4C).

### Early IFN-β administration to CS-exposed mice restored innate PRR and cytokine responses to influenza infection

**Figure 5:**
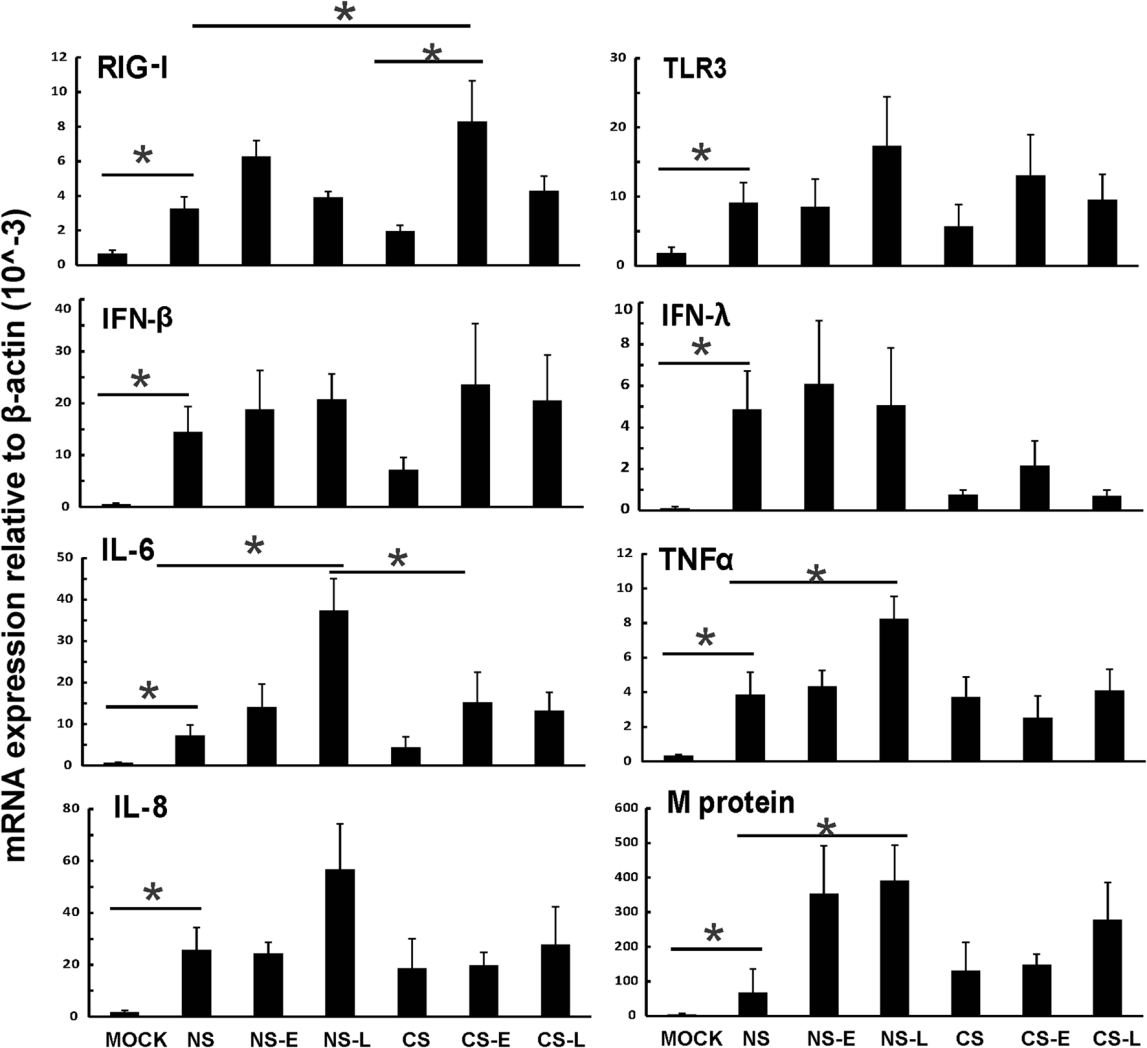
Early IFN-β administration to CS-exposed mice induced an unimpaired antiviral response to influenza infection. CS exposure, early or late IFN-β administration and IAV infection are the same as Fig. 2A. Mice were infected with 500 PFU of IAV. The mice were sacrificed at day 5 post infection and mRNA levels in the lungs were assessed by qRT-PCR and normalized by β-actin. Data are expressed as mean ± SEM (n = 5). * denotes significant difference between the two groups, *p* < 0.05.

We measured PRRs and their downstream cytokine expression during infection in all mouse groups. Mice were inoculated intranasally with a single dose of the IAV PR8 strain (500 PFU). Lung tissues and BALF were collected at 5 days after infection. mRNA or protein expression was determined by qRT-PCR or multiplex immunoassay, respectively. CS exposure suppressed RIG-I and TLR3 induction by IAV in mice compared to that in NS mice (Fig. 5 CS vs. NS). Early IFN-β administration significantly increased RIG-I mRNA induction by virus infection in CS mice (Fig. 5 CS-E vs. CS, *P* < 0.05). CS-E also restored IFN-β mRNA induction and increased IFN-λ mRNA induction by IAV infection.

**Figure 6:**
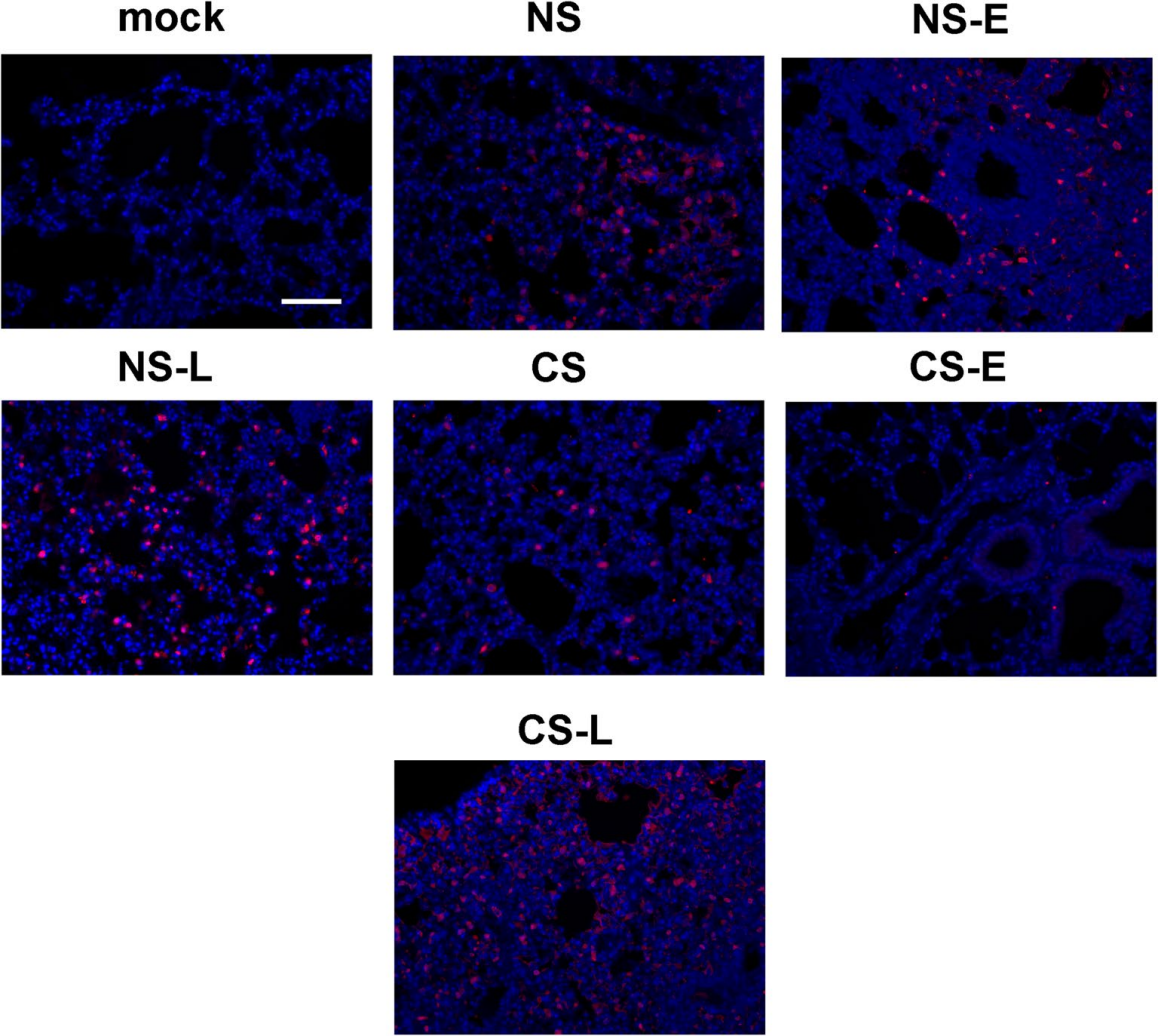
IFN-β administration altered IAV replication in mouse lung. CS exposure, early or late IFN-β administration and IAV infection of the mice were performed as in Fig. 2A. Mice were infected with 500 PFU of IAV or PBS (mock). The mice were sacrificed at day 5 post infection. Mouse lungs were processed for immunohistochemistry for detection of IAV nucleoprotein (NP, red) using an anti-NP polyclonal antibody labelled with Alexa Fluor 594. Nuclei were stained with DAPI (blue) and were shown in overlaid images with NP. The lungs of 3 mice from each treatment group were processed for immunohistochemistry, and results shown were typical for the groups. The bar represents 100 μm for all the images.

Consistent with the histopathology data, NS-L had the most robust proinflammatory cytokine IL-6, TNFα and IL-8 mRNA induction among all the groups (Fig. 5). CS-E did not change the induction of these cytokines compared to the CS group. In terms of viral RNA expression, the NS-E and NS-L groups had the most IAV M protein mRNA expression, which indicated IFN-β administration to NS mice might impair viral clearance and was unhelpful to contain the viral replication in the lung as compared to NS mice (Fig. 5 last panel). This does not occur in the CS-E group, which had decreased mortality during lethal IAV infection, as viral replication as evidenced by M protein mRNA levels was not enhanced by early IFN-β administration. In contrast in the CS-L group, M protein mRNA levels were increased, suggesting enhancement of viral replication by late administration of IFN-β. Lungs were also processed for immunohistochemistry for detection of IAV nucleoprotein (NP)^18^. The mock group showed minimal background immunofluorescence when stained for NP (Fig. 6). Viral NP expression was more widely and evenly distributed in the lungs of NS-E, NS-L and CS-L mice while the expression was more isolated in NS, CS, and CS-E mice (Fig. 6). The results suggested that virus replicated more rapidly in NS-E, NS-L and CS-L groups compared to NS, CS, and CS-E groups.

**Figure 7:**
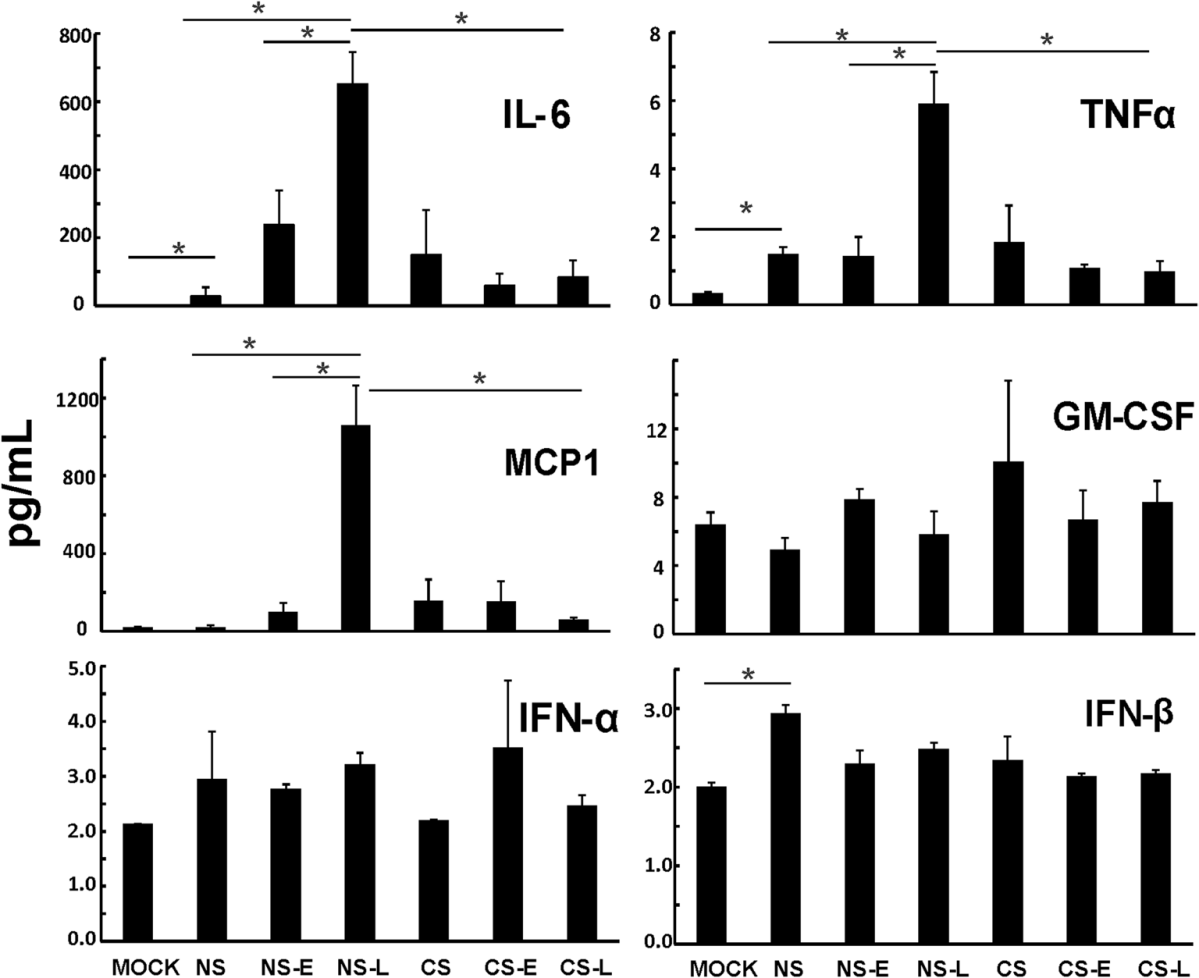
Antiviral and proinflammatory cytokine protein levels in BALF from mice. CS exposure, early or late IFN-β administration and IAV infection are the same as Fig. 2A. Mice were infected with 500 PFU of IAV. BALF were harvested at day 5 post infection. Mock treated mice were inoculated with PBS. Antiviral and proinflammatory cytokine protein levels were determined by multiplex immunoassay. Data are expressed as mean ± SEM (n ≥ 4 per group). * denotes significant difference between the two groups, *p* < 0.05.

To characterize the cytokine induction at the level of translation, we measured cytokine proteins in mouse BALF in all groups using multiplex immunoassay (Fig. 7). Again, NS-L had the most proinflammatory cytokine IL-6, TNFα and MCP-1 protein induction by IAV among all the groups. CS exposure reduced induction of IFN-α and IFN-β protein in IAV infected mice (CS vs. NS). Early IFN-β administration to CS mice appeared to enhance IFN-α protein induction by IAV. In contrast, we did not find any apparent IFN-β protein changes among any of our groups. The current study was designed to look at changes in cytokine levels just prior to animal death. Therefore, early changes in IFN-β caused by CS (day 1–4 p.i.) or by IFN-β administration were not captured. Taken together, these results demonstrate that early, but not late IFN-β administration to CS-exposed mice (CS-E) boosted the antiviral response without enhancing proinflammatory cytokine levels. In NS mice, both early and late IFN-β administration significantly enhanced pro-inflammatory cytokine (IL-6, TNFα and MCP-1) levels in mouse lungs.

## Discussion

IAV attacks the human upper respiratory tract and lung cells, and causes significant seasonal or pandemic morbidity and mortality. Autopsy of the lungs of succumbed patients always reveals diffuse alveolar disease, but viral RNA is present in only a subset of patients. The most significant histopathological findings of fatal influenza cases are pulmonary hyaline membranes and septal edema consistent with diffuse alveolar damage, tracheitis, and necrotizing bronchiolitis. Other early changes include pulmonary vascular congestion and, in some cases, alveolar hemorrhage^19^. Experimental and clinical studies have identified overly exuberant systemic inflammation as an important pathophysiologic mechanism correlating with ALI and disease severity. Inadequate viral clearance may be a less important contributor to the outcome in IAV infection^20^.

The role of IFN in acute influenza infection is complicated. Type I IFNs can have defensive antiviral effects or contribute to immunopathology^21^. Here, we tried to determine the therapeutic effects of different IFN administration times on NS and CS-exposed mice during IAV infection. Although IFN-β administration to NS mice increased mortality to IAV, early, but not late, IFN-β administration to CS-exposed mice decreased mortality during influenza infection. Comparative analyses of inflammation and ALI in these mice revealed that the host response to IAV differs significantly in pro-inflammatory activity. IFN-β administration to NS mice had the strongest pro-inflammatory responses to virus infection with an increase in differential expression of genes associated with inflammation, such as IL-6, TNF-α and IL-8. This is consisted with our current knowledge about the role of IFNs in inflammation and inflammasome activation^22^. Our results further confirmed the work by Davidson et al. which found that excessive type I IFN signaling in reaction to IAV infection can lead to uncontrolled inflammation^11^. In clinical flu cases, IFNs were robustly induced earlier at high levels^23^. Another study also demonstrated that virus-induced type I IFN-related pathways were stimulated during the initial four days of symptomatic influenza in hospitalized patients^24^. Thus, NS mice themselves have similar kinetics of IFN responses to IAV infection as do humans. Our findings suggest that IFN-β treatment, especially late IFN, to healthy NS people infected with flu would only exaggerate the IFN levels in the host and exogenous IFNs promote pro-inflammatory responses and recruit extra granulocytes and monocytes to the lung, which would contribute to worse disease outcomes.

It is a different story for CS-exposed mice or human smokers. We found that early IFN-β administration to CS mice raised IFN to an optimal level to control viral replication while imposing minimal damage on host cells. Our previous work has demonstrated that CS exposure suppressed antiviral responses to IAV infection in these mice^4,5^. Early IFN-β administration to CS mice restored the CS-suppressed RIG-I-initiated antiviral responses but did not promote excessive inflammation and ALI in the lung. The result will help design strategies for the development of IFN treatments to mitigate the adverse consequences of virus infection in immunocompromised people, such as smokers and aged. In these people, IFNs are induced less because of the suppressed immune responses of the host. On the other hand, early IFN treatment may protect the patients who are infected with weak IFN triggering viruses, such as severe acute respiratory syndrome coronavirus 2 (SARS‑CoV‑2). An in vitro study demonstrated that SARS-CoV-2 does not trigger an IFN response in primary human airway epithelial (pHAE) but is sensitive to the effects of type I and III IFNs^25^. In hospitalized coronavirus disease 2019 (COVID-19) patients, IFN production was both decreased and delayed, induced only in a portion of patients as they became critically ill^23^. Single-cell transcriptomics demonstrated weak type I IFN induction in the myeloid cells of patients with severe COVID-19, and brief expression of IFN-stimulated genes^26^. Transcriptome analyses found that patients with COVID-19 had profoundly impaired induction of type I IFNs juxtaposed to exuberant inflammatory cytokine production^23,27^. Highly impaired type I IFN activity is a hallmark of severe COVID-19^28^. Thus, exogenous early IFN treatment would modify the host innate responses to react more appropriately to the viruses, which suppress the normal IFN response in the host in a similar manner as smoking. Murine models of SARS-CoV-1 infection showed that late IFN production is associated with lung lesions and high mortality whereas early administration of IFN prevents lung damage^13^. Most importantly, one recent publication confirmed that IFN administration may be efficacious during SARS-CoV-2 infection. Intranasal administration of type I IFN reduced the viral load and lung pathology in SARS-CoV-2 infected hamsters^29^.

**Figure 8:**
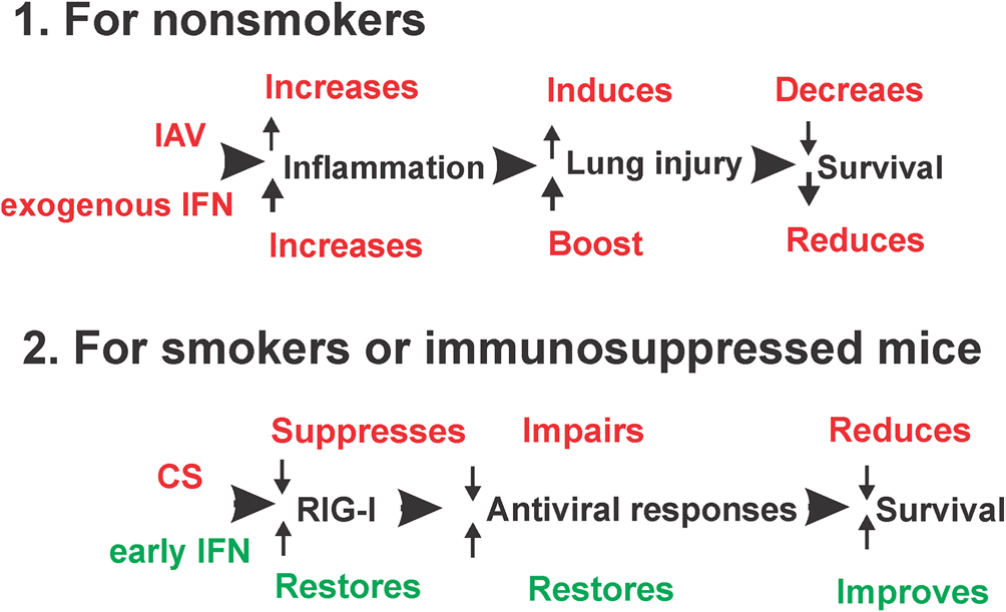
Proposed model for IFN administration to NS and CS-exposed mice during IAV infection.

Taken together, we showed here that IFN-β administration to NS mice led to uncontrolled inflammation and ALI, and decreased viral containment in the NS. Late IFN-β administration in CS mice had a similar effect. However, early IFN-β administration to CS-exposed mice played protective roles against mortality and morbidity during IAV infection (Fig. 8). It appears, therefore, that the effects of IFN-β administration during viral infection depends on the pre-existing state of the antiviral response. If the response is normal, then IFN administration may be harmful. However, if the antiviral response is impaired, administration of IFN may be helpful during viral infection. Enhancing our knowledge of pulmonary host defenses is of central importance to the development of new strategies to treat and prevent IAV infection, particularly in high-risk groups, including those exposed to CS. The timing of IFN exposition to immunosuppressed people or specific virus infection, which tend to induce weak IFN response in the host, may be crucial to limit the viral reproduction and avoid immunopathogenesis.

## Materials and methods

### Ethics statement

The Institutional Animal Care and Use Committee (IACUC) of the University of Oklahoma Health Sciences Center approved all of the protocols for the animal experiments (protocol number: 17–106-HI). The facility where this research was conducted is accredited by AAALAC. The facility operates according to the Guide for the Care and Use of Laboratory Animals, and the requirements of the Animal Welfare Act and Regulations and the Public Health Service Policy on Humane Care and Use of Laboratory Animals. All procedures were performed by personnel trained in the techniques according to IACUC guidelines. All invasive clinical procedures were performed while animals were anesthetized. In compliance with the Animal Welfare Act principles, we reduced the number of animals sacrificed by omitting control groups for which comparative effects are already known. The animals were sacrificed by overdose isofluorane. Our report follows the recommendations in the ARRIVE (Animal Research: Reporting of In Vivo Experiments) guidelines.

### Preparation of influenza virus stock and plaque assays^30^

Influenza virus, A/PR/34/8 (PR8), was passaged in Madin-Darby canine kidney (MDCK, ATCC, Manassas, VA) cells. Virus was grown in MDCK cells in DMEM/F12 with ITS + (BD Biosciences, Franklin Lakes, NJ) in the presence of TPCK-trypsin, harvested at 72 h post-infection. There was no detectable endotoxin in the final viral preparations used in the experiments as determined by limulus amebocyte lysate assay (Cambrex, Walkersville, MD). The lower limit of detection of this assay is 0.1 EU/ml or approximately 20 pg/ml LPS. The virus was titered by plaque assay in MDCK cells, aliquoted and stored at − 80 °C.

### Whole-body CS exposure^5^

C57BL/6 mice were exposed to the smoke of 1R6F reference cigarettes (University of Kentucky, Lexington, KY) for 4 h per day. Mice receiving CS were gradually brought up to the target exposure over a period of 2 weeks, and treated 5 days/week for 6 weeks. Treatment was administered by placing mice in a Plexiglas smoking chamber (Teague Enterprises, Davis, CA). Smoke exposure was standardized to total suspended particles = 90 mg/m^3^, 11% mainstream and 89% sidestream smoke in the chamber of the machine. The exposure level was assessed by measuring serum cotinine, a nicotine metabolite, at 1 h after exposure (Cotinine ELISA Kit, GenWay Biotech Inc.). After six-week exposure, the average serum cotinine was 513 ± 256 ng/ml, near levels in human cigarette smokers^31^. “Nonsmoking” (NS) treatment groups were conducted for the same periods of time, but mice were exposed to filtered room air.

### IFN-β administration and Influenza virus infection

IFN-β administration and IAV infection were performed under isoflurane anesthesia. Animals that died during anesthesia were removed from subsequent calculations of mortality. The mice were treated with IFN-β (2000 U) intranasally in a total volume of 50 μl in 0.1%BSA/PBS. Control groups were sham treated with a single dose of intranasal sterile 0.1%BSA/PBS solution. IAV PR8 stock was diluted in PBS to make lethal doses of virus. These virus doses (50 µl solution) were administered by intranasal instillation as the animal was held in a vertical position while sedated. Two doses were chosen to achieve an LD_50_ in NS (1000 PFU), or CS (500 PFU) animals. The mock group was sham inoculated with an equal volume of PBS. Mice were monitored daily for 16 days for clinical symptoms (shaking, inactivity and piloerection) and their weight was recorded daily.

### Multiplex immunoassay

Cytokine protein levels in the bronchoalveolar lavage fluids (BALF) were determined by multiplex immunoassay (Eve Technologies, Calgary, AB, Canada). All samples were twofold diluted in 1% triton X-100 (final) for decontamination.

### Measurement of mRNA expression by quantitative real-time PCR (qRT-PCR)

Total RNA from lung was extracted using a modified TRIzol (Invitrogen, Carlsbad, CA) protocol and spectrophometrically quantitated. The integrity of RNA was verified by formaldehyde agarose gel electrophoresis. Equal amounts (1 µg) of RNA from each sample were reverse-transcripted into cDNA with oligo (dT) SuperScript II First-Strand Synthesis System for RT-PCR (Invitrogen, Carlsbad, CA). Gene specific primers for mouse PRRs, cytokines and the β-actin housekeeping genes were used. The primers’ sequences were the same as in our earlier publication^6^. qRT-PCR was performed using 100 ng sample RNA and SYBR Green (Quanta Biosciences, Gaithersburg, MD) in a Bio-Rad CFX96™ Touch Real-Time PCR Detection System. Results were calculated and graphed from the ΔCT of target gene and normalizer, β-actin.

### Histological and immunohistochemistry analysis of mouse lung

At day 5 after IAV infection, mice were sacrificed and lungs were fixed in 4% paraformaldehyde in PBS at room temperature for 30 min, and were then embedded in paraffin. Fixed tissue was hematoxylin and eosin (H & E) stained to assess inflammation and fibrosis. Histopathologic scoring of the cardinal features of IAV infection was evaluated using a modified histological scoring system in a previous publication^17^. Briefly, four easily identifiable pathological processes were chosen in order to grade, semiquantitatively, on a scale of 0–4: alveolar and interstitial oedema; haemorrhage; margination; and infiltration of inflammatory cells and formation of bronchiolitis. A score of 0 represented normal lungs; 1 represented mild; 2 moderate; 3 severe; and 4 very severe histopathological changes. The results of histopathological changes were expressed as mean ± SEM (three sections from each lung, 5 lungs per group) on each group. Sections (3–5 μm) were mounted on glass slides and immuno-probed with an anti-NP polyclonal antibody^18^. After washing, the sections were probed with a donkey anti-rabbit secondary antibody conjugated to Alexa Fluor 594 (BD/Molecular Probes). Transmitted light and fluorescent microscopy images were obtained from an Olympus BX51 microscope running Cellsens imaging software (Olympus Corp., Center Valley, PA).

### Statistical analysis

Statistical significance was determined by one-way ANOVA with Student–Newman–Keuls post hoc correction for multiple comparisons. Survival rate significance was determined by Fisher’s exact test. For RT-PCR results, the *p* value was calculated from the ΔCt values from different experimental groups. Significance was considered as *p* < 0.05.

### Ethics statement

The animal research was approved by the Institutional Animal Care and Use Committee of the University of Oklahoma Health Sciences Center (Approval number 101089-14-152-HI).

## Supplementary Information

Supplementary Information.

## Data Availability

The original contributions presented in the study are included in the article/Supplementary Material. Further inquiries can be directed to the corresponding authors.
