# Supplementary material for: Early IFN-β administration protects cigarette smoke exposed mice against lethal influenza virus infection without increasing lung inflammation
Source: Sci Rep. 2022 Mar 8;12:4080. doi: 10.1038/s41598-022-08066-7 (PMC8902729; doi:10.1038/s41598-022-08066-7)

**Supplementary Materials**

**Fig S1. The typical lung histopathology after IAV infection as shown by haematoxylin–eosin staining.** Each mouse was infected with 500 PFU of IAV PR8. Animals were sacrificed at 5 days after infection and lung tissue was harvested. Lung tissue sections prepared from the infected mice were fixed, processed and stained with haematoxylin–eosin. **Panel A**. Bronchi (arrows) and bronchioles (arrowheads) exhibit severe inflammation characterized by epithelial cell necrosis and sloughing with partial to complete airway obstruction by intact and degenerate neutrophils and cellular debris, Bar=100µm. **Panel B**. Airways and alveolar spaces are partially occluded by red blood cells (hemorrhage), Bar=50µm. **Panel C**. Margination of neutrophils is present within septal capillaries and pulmonary venules (arrowheads) with extravasation into neighboring alveolar compartments (arrows), Bar=20µm. **Panel D**. Margination of neutrophils was most conspicuous in mice exhibiting marked extravasation of neutrophils into airways (arrows) and alveolar spaces (arrowheads), Bar=50 µm. **Panel E**. Alveolar spaces are occluded by pale, homogeneous eosinophilic material characteristic of edema fluid, Bar=100µm.

Supplemental Figure 1


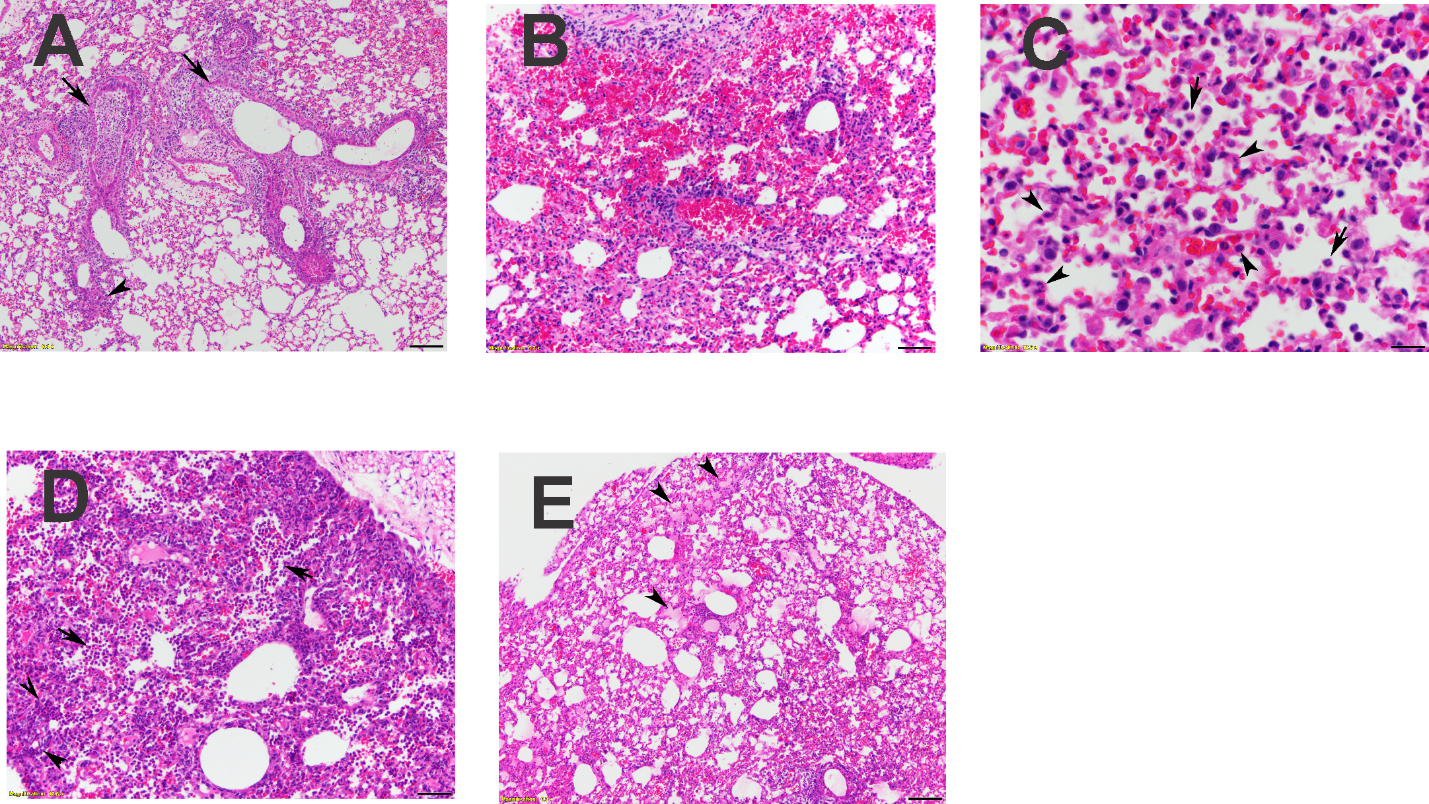

Supplement: Supplementary file 1 — Supplementary Information. [file 41598_2022_8066_MOESM1_ESM.docx]
